# The expression and mutation of *BMPR1B* and its association with litter size in small-tail Han sheep (*Ovis aries*)

**DOI:** 10.5194/aab-64-211-2021

**Published:** 2021-05-28

**Authors:** Yu-Liang Wen, Xiao-Fei Guo, Lin Ma, Xiao-Sheng Zhang, Jin-Long Zhang, Sheng-Guo Zhao, Ming-Xing Chu

**Affiliations:** 1 Key Laboratory of Animal Genetics, Breeding and Reproduction of Ministry of Agriculture and Rural Affairs, Institute of Animal Science, Chinese Academy of Agricultural Sciences, Beijing 100193, China; 2 College of Animal Science and Technology, Gansu Agricultural University, Lanzhou 730070, China; 3 Tianjin Institute of Animal Sciences, Tianjin 300381, China

## Abstract

Previous studies have shown that *BMPR1B* promotes follicular development and ovarian granulosa cell proliferation, thereby affecting ovulation in mammals. In this study, the expression and polymorphism of the *BMPR1B* gene associated with litter size in small-tail Han (STH) sheep were determined. The expression of *BMPR1B* was detected in 14 tissues of STH sheep during the follicular phase as well as in the hypothalamic–pituitary–gonadal (HPG) axis of monotocous and polytocous STH sheep during the follicular and luteal phases using quantitative polymerase chain reaction (qPCR). Sequenom MassARRAY^®^ single nucleotide polymorphism (SNP) technology was also used to detect the polymorphism of SNPs in seven sheep breeds. Here, *BMPR1B* was highly expressed in hypothalamus, ovary, uterus, and oviduct tissue during the follicular phase, and *BMPR1B* was expressed significantly more in the hypothalamus of polytocous ewes than in monotocous ewes during both the follicular and luteal phases (P<0.05). For genotyping, we found that genotype and allele frequencies of three loci of the *BMPR1B* gene were extremely significantly different (P<0.01) between the monotocous and polytocous groups. Association analysis results showed that the g.29380965A>G locus had significant negative effects on the litter size of STH sheep, and the combination of g.29380965A>G and *FecB* (Fec – fecundity and B – Booroola; A746G) at the *BMPR1B* gene showed that the litter size of AG–GG, AA–GG, and GG–GG genotypes was significantly higher compared with other genotypes (P<0.05). This is the first study to find a new molecular marker affecting litter size and to systematically analyze the expression of *BMPR1B* in different fecundity and physiological periods of STH sheep.

## 1 Background

Lambing is an important economic trait of sheep and is closely related to the economic benefits of sheep breeding. However, as it is a complex threshold trait, it is very inefficient if it relies on traditional breeding methods (Miao and Luo, 2013). The genes affecting the prolificacy of sheep have received much attention from researchers since the 1980s (Tang et al., 2018). Bone morphogenetic protein receptor 1B (*BMPR1B*), as the receptor of the bone morphogenetic protein (BMP) family, has been identified as a major gene affecting the litter size in sheep (Chu et al., 2011; Wozney et al., 1988; Juengel et al., 2013). Studies have shown that *BMPR1B* is widely expressed in mammalian ovaries, including humans, mice, sheep, and cattle, and plays an important role in early embryonic development, synthesis of the extracellular matrix, and regulation of ovarian development in sheep (Souza et al., 2001; Elizabeth et al., 2010; Juengel et al., 2013; Khalaf et al., 2013; Selvaraju et al., 2013). Mice deficient in the *BMPR1B* gene have been shown to experience infertility, as *BMPR1B* deficiency affects the proliferation of cumulus granulosa cells and reduces the aromatase content, causing irregular estrous cycles (Mishina et al., 1995; Sun and Li, 2013).

*BMPR1B* is a bone morphogenetic protein type 1 receptor containing 11 exons that encoding 502 amino acids with a coding region of 1509 bp. Its extracellular region contains approximately 150 amino acids, and the intracellular region of the first 30 amino acids of the kinase domain contains a unique GS (glycine- and serine-rich sequence) domain and a 55 ku glycoprotein (Emmerson et al., 2018; Li et al., 2018). *FecB* (Fec – fecundity and B – Booroola; A746G) missense mutation in the *BMPR1B* gene causes amino acid changes (Q249R), thereby increasing ovulation and lambing in sheep (Souza et al., 2001). This mutation has an additive effect on ovulation in sheep – that is, the increase in the ovulation increases by one copy (Piper et al., 1985; Guo et al., 2018; Chong et al., 2019). A previous study has shown that *BMPR1B* mutation increases the signal intensity of the signal transduction process to downstream receptors, leading to premature follicles and increased ovulation (Guo et al., 2018). The *FecB* mutation is widely distributed. In addition to Booroola Merino sheep, the mutation, which can improve twin production (El Seedy et al., 2017; Darwish, 2018), has been detected in Kendrapada sheep (Mahdavi et al., 2014) in India, Javanese sheep (Kumar et al., 2008) in Indonesia, Kalehkoohi sheep (Mahdavi et al., 2014) in Iran, and Hu sheep (Feng et al., 2006) in China. With deep research into high-fecundity genes in sheep, the *FecB* gene has been increasingly applied to the cultivation of new sheep varieties. For example, Chen et al. (2015) used the *FecB* effect to cross small-tail Han (STH) sheep with Dorper sheep, and the average litter size in the hybrid offspring was significantly higher than in Dorper sheep (P<0.05). CRISPR/Cas9 technology has also been applied to sheep embryos, establishing a technical basis for editing the sheep *BMPR1B* gene (Zhang et al., 2017; Zhou et al., 2018; Rui et al., 2019). Given the significance of the *BMPR1B* gene, the discovery of other SNPs is particularly important. The whole genomes were re-sequenced in 10 sheep breeds in the early stage of our study. Three SNPs were screened based on the Oar_3.1 reference genome (Pan et al., 2018). Therefore, we hypothesized that these SNPs may be related to the litter size in STH sheep. Thus, the expression pattern of the *BMPR1B* gene in STH sheep during different fecundity (polytocous and monotocous) and different physiological (follicular and luteal) periods were detected using quantitative polymerase chain reaction (qPCR). The Sequenom MassARRAY^®^ single nucleotide polymorphism (SNP) technique was used to genotype the three SNPs in a large group, and association analysis was then conducted with respect to the litter size of STH sheep. The study focused on the *BMPR1B* gene expression, new molecular markers affecting litter size, and how these mutations provided new insights into the control of ovarian function in STH sheep.

## 2 Materials and methods

### 2.1 Animal processing

A total of 12 STH ewes (*FecB* ++ genotype) were randomly selected from Yuncheng (Yuncheng County, Shandong, China): six monotocous and six polytocous ewes according to lambing records. All ewes were healthy and approximately 3 years old. The abovementioned 12 test sheep were injected with vaginal progesterone embolization (controlled internal drug releasing, CIDR) for simultaneous estrus. After 12 d, follicular development and ovulation were observed via laparoscopy to determine the estrus period and sampling time; specifically, the follicular phase was 45 h after withdrawal, and the luteal phase was 10 d after withdrawal. A total of 14 tissue samples (heart, liver, spleen, lung, kidney, thyroid, adrenal gland, brain, cerebellum, hypothalamus, pituitary, ovary, uterus, and oviduct) were collected from each six follicular-phase (three monotocous and three polytocous) ewes and six luteal-phase (three monotocous and three polytocous) ewes after the 12 ewes were slaughtered. All selected samples were stored at -80 ${}^{\circ }$C for RNA extraction.

A total of 768 blood samples collected from seven sheep breeds were used for DNA extraction, including three monotocous breeds (384 STH sheep, 83 Hu sheep, and 68 Cele black sheep) and four polytocous breeds (80 Prairie Tibetan sheep, 60 Suffolk sheep, 70 Sunite sheep, and 23 Tan sheep) (Table 1).

**Table 1:** Information on seven sheep breeds for genotyping in the present study.

Breed	Group	Number	Type	District
Small-tail Han sheep	Polytocous	384	Year-round breeding	Southwest region, Shandong Province, China
Hu sheep	Polytocous	83	Year-round breeding	Xuzhou, Jiangsu Province, China
Cele black sheep	Polytocous	68	Year-round breeding	Cele, Xinjiang Uygur Autonomous Region, China
Sunite sheep	Monotocous	70	Seasonal breeding	Wulatezhongqi, Bayannaoer, Inner MongoliaAutonomous Region, China
Prairie Tibetan sheep	Monotocous	80	Seasonal breeding	Dangxiong, Tibet Autonomous Region, China
Suffolk sheep	Monotocous	60	Seasonal breeding	Beijing Aoxin Stud Farm Co. Ltd., located in Shunyi District, Beijing, China
Tan sheep	Monotocous	23	Seasonal breeding	Yanchi, Ningxia Hui Autonomous Region, China

### 2.2 Total DNA and RNA preparation

Blood DNA and tissue RNA were extracted using a DNA extraction kit (TIANGEN, Beijing, China) and an RNA extraction kit (TIANGEN, Beijing, China), respectively, with TRIzol (Invitrogen Inc., Carlsbad, CA, USA). The quantity and quality of total DNA and RNA were monitored to ensure that they met the requirements for subsequent experiments; the reader is referred to Chen et al. (2020) for details on the specific methods utilized.

### 2.3 Primer design

The primers for the sheep *BMPR1B* and *RPL19* genes were designed based on their sequences in GenBank (Table 2). *RPL19* (accession no. XM_012186026.1) was used as an internal control to normalize the threshold cycle (Ct) values. Primers were synthesized by Beijing Tianyi Biotechnology (Beijing, China).

**Table 2:** Primers used in the present study.

Gene	GenBank ID	Primer sequence (5${}^{\prime }$ → 3${}^{\prime }$)	Product size (bp)	$T_{m}$ (${}^{\circ }$C)	Application
*BMPR1B*	NM_001009431.1	F: TGACGGACCTATACACCACA R: GTACCGAGGTCTGGCTTCTT	121	60	qPCR
*RPL19*	XM_012186026.1	F: AATGCCAATGCCAACTC R: CCCTTTCGCTACCTATACC	151	60	Reference gene

### 2.4 qPCR

The first strand of cDNA was prepared following the instructions of the PrimeScript™ RT Reagent Kit (TaKaRa Bio Inc., Dalian, China). Real-time quantitative polymerase chain reaction (qPCR) amplification was performed in a 20 µL reaction mixture, containing 10 µL SYBR Premix (TaKaRa, Dalian, China), 0.4 µL of each forward and reverse primer (20 ng/µL), 7.2 µL ddH${}_{2}$O, and 2 µL cDNA (200 ng/µL). The reaction conditions were as follows: initial denaturation at 95 ${}^{\circ }$C for 5 min, denaturation at 95 ${}^{\circ }$C for 10 s, annealing at 60 ${}^{\circ }$C for 30 s, and extension at 72 ${}^{\circ }$C for 30 s (40 cycles). The relative expression levels of *BMPR1B* and *RPL19* mRNA were analyzed using the $2^{-\Delta \Delta \mathrm{Ct}}$ method (Livak and Schmittgen, 2001).

### 2.5 Genotyping

In the early stage of our study, three SNPs (g.29380950G>A, g.29380965A>G, and g.29401381C>T) of the *BMPR1B* gene were obtained. The above SNPs were then typed using the Sequenom MassARRAY^®^ SNP technique. The 768 blood DNA (40–80 ng/µL, 20 µL) samples from the different breeds were used for genotyping (Table 1); the reader is referred to Zhou et al. (2018) for details on the typing step utilized.

### 2.6 Statistical analysis

Statistical analyses were performed using an analysis of variance (ANOVA), followed by a Fisher's least significant difference test as a multiple comparison test in SPSS 19.0 software (IBM, Armonk, New York, USA). The adjusted linear model, $y_{ijkl}=\mu$ + Genotype${}_{i}$ + $S_{k}+e_{ijkl}$, in which $y_{ijkl}$ is the trait observation, μ is the overall average, Genotype${}_{i}$ is the genotype effect, $S_{k}$ is the season effect, and $e_{ijkl}$ is the random error, was applied to determine the association between genotypes and the litter size in STH sheep. It is assumed that random errors ($e_{ijkl}$) are independent of each other and obey the $N(0,\sigma^{2})$ distribution. The allele and genotype frequency, polymorphism information content (PIC), heterozygosity ($H_{e}$), number of effective alleles ($N_{e}$), and $\chi^{2}$ (chi-square) value were calculated using the data from genotyping; ewe populations with P>0.05 (based on the $\chi^{2}$ test) were considered to be in Hardy–Weinberg equilibrium. All experimental data are presented as mean ± SE (standard error of the mean): P≤0.05 indicates that the difference was significant; P≤0.01 indicates that the difference was extremely significant.

## 3 Results

### 3.1 Expression levels of *BMPR1B* in different tissues during the follicular phase

**Figure 1:**
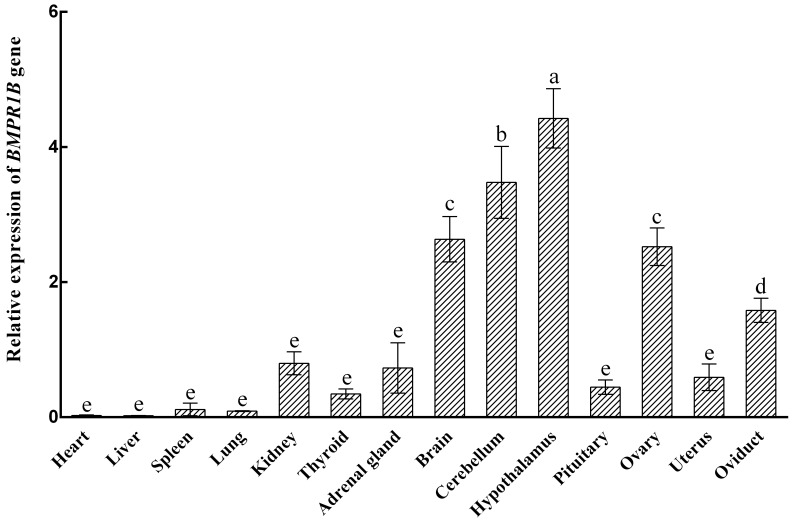
Relative expression of the *BMPR1B* gene in various tissue types in small-tail Han sheep in the follicular phase. Different letters represent a significant difference (P<0.05).

The expression characteristics of the *BMPR1B* gene are shown in Fig. 1. The *BMPR1B* gene was highly expressed in the brain and in reproduction-related tissues. Taking the expression of the *BMPR1B* gene in the pituitary as a reference, the expression levels in the brain, cerebellum, hypothalamus, ovary, and oviduct were 5.92 times (P<0.001), 7.82 times (P<0.001), 9.95 times (P<0.001), 5.67 times (P<0.001), and 3.56 times (P=0.004) higher, respectively. The expression of *BMPR1B* in the uterus was 1.33 times higher than that of pituitary tissue, but this difference was not significant (P>0.05).

### 3.2 Expression of *BMPR1B* in the HPG axis tissues during different physiological periods

**Figure 2:**
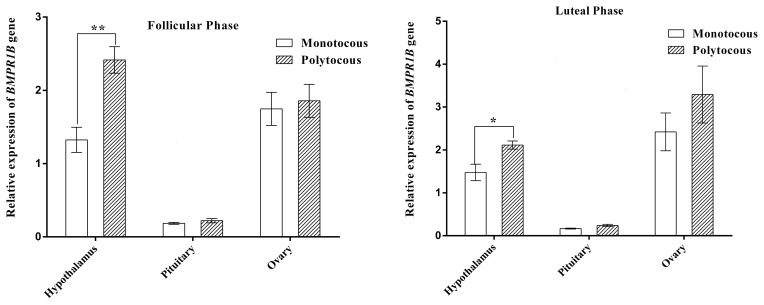
Relative expression of the *BMPR1B *gene in HPG axis tissues of small-tail Han sheep. “*” denotes significant differences, and “**” denotes extremely significant differences.

As shown in Fig. 2, *BMPR1B* expression in the hypothalamus of the polytocous group in the follicular and luteal phases was significantly higher than expression in the monotocous group (P<0.05). The expression of the polytocous group in the pituitary and ovarian tissues in the follicular and luteal phases was higher than that of the monotocous group but did not reach a significant level (P>0.05).

### 3.3 Polymorphism of the *BMPR1B* gene

**Figure 3:**
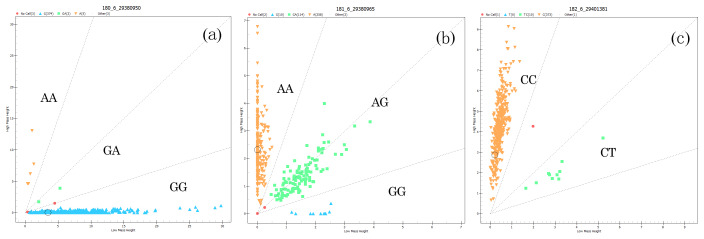
Genotyping in three loci of *BMPR1B*. The upper-left area indicates the AA genotype **(a)** with 5 individuals, the AA genotype **(b)** with 258 individuals, and the CC genotype **(c)** with 373 individuals; the middle area indicates the GA genotype **(a)** with 2 individuals, the AG genotype **(b)** with 114 individuals, and the CT genotype **(c)** with 10 individuals; the lower-right area indicates the GG genotype **(a)** with 374 individuals and the GG genotype **(b)** with 10 individuals.

The genotyping results of the g.29380950G>A, g.29380965A>G, and g.29401381C>T loci of *BMPR1B* are shown in Fig. 3. For g.29380950G>A (Fig. 3a), for example, there are three genotypes: wild homozygous AA, heterozygous GA, and mutant homozygous GG. For the g.29401381C>T locus, in contrast, there are only two genotypes: homozygous CC and heterozygous CT (Fig. 3c).

### 3.4 Population genetic analysis of SNPs in the *BMPR1B* gene

Three SNPs (g.29380950G>A, g.29380965A>G, and g.29401381C>T) in *BMPR1B* were detected in monotocous and polytocous sheep breeds (Table 3) and for seven sheep breeds (Table 4). The allele frequencies of the g.29380950G>A, g.29380965A>G, and g.29401381C>T loci between monotocous and polytocous breeds were significantly different (P<0.01). The difference in the genotype frequency of the g.29380950G>A locus between monotocous and polytocous sheep breeds reached an extremely significant level (P<0.001), and the difference in the genotype frequency of the g.29401381C>T locus reached a significant level (P<0.05). The difference in the g.29380965A>G locus genotype frequency between monotocous and polytocous sheep breeds, in contrast, did not reach a significant level (P=0.97).

**Table 3:** Genotype and allele frequencies of three SNPs of the *BMPR1B* gene in monotocous and polytocous sheep.

Locus	Genotype	Genotype	Genotype	$\chi^{2}$ test	Allele	Allele	Allele	$\chi^{2}$ test
		frequency in	frequency in	(P value)		frequency in	frequency in	(P value)
		monotocous	polytocous			monotocous	polytocous	
		sheep (no.)	sheep (no.)			sheep	sheep	
g.29380950G>A	GG	0.90 (209)	0.99 (525)		G	0.94	0.99	
	GA	0.07 (16)	0.00 (2)	0.00	A	0.06	0.01	0.00
	AA	0.03 (7)	0.01 (5)					
g.29380965A>G	AA	0.73 (159)	0.73 (391)		A	0.85	0.86	
	AG	0.25 (55)	0.25 (131)	0.97	G	0.15	0.14	0.01
	GG	0.02 (5)	0.02 (11)					
g.29401381C>T	CC	0.94 (217)	0.98 (521)		C	0.97	0.99	
	CT	0.06 (15)	0.02 (13)	0.02	T	0.03	0.01	0.01
	TT	0.00 (0)	0.00 (0)					

P<0.05 denotes significant differences, and P<0.01 denotes extremely significant differences.

The allele frequency, genotype frequency, PIC, $H_{e}$, $N_{e}$, and $\chi^{2}$ test results from population genetic analysis for three SNPs in the seven sheep breeds are listed in Table 4. The g.29380950G>A locus showed moderate polymorphism (0.25 < PIC < 0.5) in the Tan sheep breed and low polymorphism in the other sheep breeds (PIC < 0.25); the g.29380965A>G locus showed moderate polymorphism (0.25 < PIC < 0.5) in STH sheep, Sunite sheep, and Suffolk sheep, whereas it displayed low polymorphism in other breeds (PIC < 0.25); the g.29401381C>T locus showed low polymorphism in all seven sheep breeds (PIC < 0.25). The $\chi^{2}$ test results showed that the g.29380950G>A locus was not in Hardy–Weinberg equilibrium (P<0.05) except for in the Tan and Prairie Tibetan sheep breeds; the g.29380965A>G locus was in Hardy–Weinberg equilibrium in the seven sheep breeds (P>0.05); the g.29401381C>T locus was in Hardy–Weinberg equilibrium in all sheep breeds (P>0.05) except for Hu sheep.

**Table 4:** Population genetic analysis of different SNPs of the *BMPR1B* gene in seven sheep breeds.

Locus	Breed	Genotype			Allele		Polymorphism	Heterozygosity	Effective	$\chi^{2}$ test
		frequency			frequency		information	($H_{e}$)	number of	(P value)
							content (PIC)		alleles ($N_{e}$)	
		GG	GA	AA	G	A				
g.29380950G>A	Small-tail Han sheep	0.98 (374)	0.01 (3)	0.01 (5)	0.98	0.02	0.03	0.03	1.03	0.00
	Hu sheep	1.00 (83)	0.00 (0)	0.00 (0)	1.00	0.00	0.00	0.00	1.00	0.00
	Cele black sheep	1.00 (68)	0.00 (0)	0.00 (0)	1.00	0.00	0.00	0.00	1.00	0.00
	Tan sheep	0.65 (15)	0.22 (5)	0.13 (3)	0.76	0.24	0.30	0.36	1.57	0.05
	Sunite sheep	0.93 (65)	0.04 (3)	0.03 (2)	0.95	0.05	0.09	0.10	1.10	0.00
	Prairie Tibetan sheep	0.91 (73)	0.08 (6)	0.01 (1)	0.95	0.05	0.09	0.10	1.10	0.06
	Suffolk sheep	0.95 (56)	0.03 (2)	0.02 (1)	0.97	0.03	0.06	0.07	1.07	0.00
		AA	AG	GG	A	G				
g.29380965A>G	Small-tail Han sheep	0.67 (258)	0.30 (114)	0.03 (10)	0.82	0.18	0.25	0.29	1.41	0.54
	Hu sheep	0.96 (80)	0.04 (3)	0.00 (0)	0.98	0.02	0.03	0.04	1.04	0.87
	Cele black sheep	0.78 (53)	0.20 (14)	0.02 (1)	0.88	0.12	0.19	0.21	1.26	0.95
	Tan sheep	0.76 (16)	0.24 (5)	0.00 (0)	0.88	0.12	0.19	0.21	1.27	0.54
	Sunite sheep	0.66 (40)	0.33 (20)	0.01 (1)	0.82	0.18	0.25	0.30	1.42	0.39
	Prairie Tibetan sheep	0.84 (67)	0.16 (13)	0.00 (0)	0.92	0.08	0.14	0.15	1.18	0.43
	Suffolk sheep	0.63 (36)	0.30 (17)	0.07 (4)	0.78	0.22	0.28	0.34	1.52	0.33
		CC	CT	TT	C	T				
g.29401381C>T	Small-tail Han sheep	0.97 (373)	0.03 (10)	0.00 (0)	0.99	0.01	0.03	0.03	1.03	0.80
	Hu sheep	1.00 (83)	0.00 (0)	0.00 (0)	1.00	0.00	0.00	0.00	1.00	0.00
	Cele black sheep	0.96 (65)	0.04 (3)	0.00 (0)	0.98	0.02	0.04	1.05	0.85	0.85
	Tan sheep	0.70 (16)	0.30 (7)	0.00 (0)	0.85	0.15	0.22	0.26	1.35	0.39
	Sunite sheep	0.94 (66)	0.06 (4)	0.00 (0)	0.97	0.03	0.05	0.06	1.06	0.81
	Prairie Tibetan sheep	0.96 (77)	0.04 (3)	0.00 (0)	0.98	0.02	0.04	0.04	1.04	0.86
	Suffolk sheep	0.98 (58)	0.02 (1)	0.00 (0)	0.99	0.01	0.02	0.02	1.02	0.95

P<0.05 denotes significant differences, and P<0.01 denotes extremely significant differences.

### 3.5 Association between three loci of *BMPR1B* and litter size in small-tail Han sheep

The results revealed that the g.29380965A>G locus was significantly correlated with litter size in STH sheep (Table 5) and that the litter size of ewes with AA and AG genotypes was higher than that of ewes with the GG genotype (P<0.05). As for the combination of the g.29380965A>G locus and the *FecB* genotype (Table 6), the composite genotype was significantly correlated with litter size in STH sheep (P<0.05): the litter sizes of ewes with AG–GG, AA–GG, and GG–GG genotypes were significantly higher than other combination genotypes (P<0.05).

**Table 5:** Analysis of different genotypes of *BMPR1B* and litter size in small-tail Han sheep.

Locus	Genotype	First parity		Second parity		Third parity	
		No. of	Litter size	No. of	Litter size	No. of	Litter size
		individuals		individuals		individuals	
g.29380950G>A	GG	372	1.87 ± 0.03	223	2.11 ± 0.05	89	2.22 ± 0.08
	GA	4	2.17 ± 0.38	4	2.23 ± 0.38	4	2.25 ± 0.37
	AA	4	2.20 ± 0.30	4	2.45 ± 0.38	4	2.50 ± 0.37
g.29380965A>G	AA	256	1.98 ± 0.04${}^{a}$	146	2.25 ± 0.06${}^{a}$	61	2.60 ± 0.09${}^{a}$
	AG	114	1.70 ± 0.06${}^{a}$	75	1.93 ± 0.08${}^{a}$	26	2.35 ± 0.14${}^{a}$
	GG	10	1.10 ± 0.21${}^{b}$	6	1.17 ± 0.30${}^{b}$	3	1.47 ± 0.41${}^{b}$
g.29401381C>T	CC	370	1.88 ± 0.03	224	2.12 ± 0.05	88	2.42 ± 0.08
	CT	10	1.60 ± 0.21	4	1.85 ± 0.38	4	2.20 ± 0.37
	TT	–	–	–	–	–	–

Note that different lowercase letters in the same column represent a significant difference (P<0.05).

**Table 6:** Analysis of combined genotypes of *BMPR1B* (g.29380965A>G) and *FecB* and litter size in small-tail Han sheep.

Genotype	First parity		Second parity		Third parity	
	No. of	Litter size	No. of	Litter size	No. of	Litter size
	individuals		individuals		individuals	
GG-AA	8	1.00 ± 0.19${}^{b}$	6	1.17 ± 0.25${}^{c}$	3	1.37 ± 0.36${}^{b}$
AG-AA	27	1.04 ± 0.10${}^{b}$	16	1.19 ± 0.15${}^{c}$	4	1.40 ± 0.32${}^{b}$
AA-AA	24	1.09 ± 0.11${}^{b}$	10	1.20 ± 0.19${}^{c}$	3	1.53 ± 0.36${}^{b}$
AA-AG	91	2.00 ± 0.06${}^{a}$	51	2.20 ± 0.09${}^{b}$	20	2.50 ± 0.14${}^{a}$
AG-AG	84	1.89 ± 0.06${}^{a}$	58	2.12 ± 0.08${}^{b}$	23	2.20 ± 0.13${}^{a}$
GG-AG	5	1.86 ± 0.24${}^{a}$	–	–	–	–
GG-GG	5	2.00 ± 0.24${}^{a}$	–	–	–	–
AA-GG	143	2.39 ± 0.04${}^{a}$	85	2.53 ± 0.07${}^{a}$	38	2.76 ± 0.10${}^{a}$
AG-GG	3	2.33 ± 0.31${}^{a}$	4	2.46 ± 0.31${}^{a}$	4	2.52 ± 0.32${}^{a}$

Note that different lowercase letters in the same column represent a significant difference (P<0.05).

## 4 Discussion

### 4.1 Effect of *BMPR1B* gene expression on mammalian reproduction

*BMPR1B* (*FecB*) is one of the major fecundity genes in female reproduction and plays a major role in the development of follicles and the proliferation of ovarian granulosa cells in sheep (Shimasaki et al., 1999; Chu et al., 2007; Yao et al., 2019; Tao et al., 2019; Abdurahman et al., 2019). Previous reports have found that *BMPR1B* belongs to the type I receptors of BMPs (Aquino et al., 2017), which figure prominently in the proliferation of primordial germ cells (PGCs) (Nermin et al., 2018; Yi et al., 2001) and in the production and secretion of reproductive hormone (Richards and Pangas, 2010; Isaacs et al., 1995) by binding specifically to different types of ligands (Ikeda et al., 2016). Previous studies have found that *BMPR1B* is widely expressed in the brain and reproduction-related tissues of mammals (Goyal et al., 2017; Foroughinia et al., 2017) and is moderately expressed in heart, liver, spleen, lung, and muscle tissue (Tang et al., 2018; Ciller et al., 2016). In this study, *BMPR1B* was found to be expressed in all selected STH tissue types and was highly expressed in the brain, cerebellum, hypothalamus, ovary, and oviduct. This shows that it may play a major role in the normal physiological functions of various organs.

The expression of *BMPR1B* in the hypothalamic–pituitary–gonadal (HPG) axis was higher in the polytocous group than in the monotocous group in the follicle and luteal phases, reaching a significant level in the hypothalamus in the follicle phase and an extremely significant level in the luteal phase. This observation was identical to a previous study comparing high-fecundity and low-fecundity ewes (Xu et al., 2010; Goyal et al., 2017). Tang et al. (2018) explored the expression of *BMPR1B* in the HPG axis tissues using three genotypes (BB, B+, and ++ genotypes of *FecB*) and found that *BMPR1B* expression was significantly higher in the hypothalamus and pituitary in the BB group compared with the B+ and ++ groups. The *BMPR1B* gene has also been extensively studied in other species and was found to be more highly expressed in the ovary of a highly prolific goat breed (Jintang black goat) than in that of a breed with a low reproductive output (Tibetan goat) (Pan et al., 2015). In terms of embryonic development, *BMPR1B* has been found to play an important role in mouse embryonic development, and *BMPR1B* defects in mice have been shown to cause infertility (Mishina et al., 1995). In addition, *BMPR1B* defects affect the proliferation of cumulus granulosa cells and reduce the aromatase content, causing mice to exhibit an irregular estrous cycle (Yi et al., 2001; Sun and Li, 2013).

*BMPR1B* also plays an important role in follicular development as well as the synthesis and secretion of reproductive hormones. In buffalo ovarian follicular cells, according to the diameter of the follicle and the content of E${}_{2}$ (Estradiol), the follicle cells are divided into dominant follicles (D > 13 mm, E${}_{2}>180$ ng/mL) and other cells, and the expression of *BMPR1B* in the granulosa cells and ovarian membrane cells of dominant follicles is 1.5–2.0 times higher than that of other follicles (Rajesh et al., 2018; Elizabeth et al., 2010). Costa et al. (2012) found that the expression of the *BMPR1B* gene in a 0.2 mm follicle was higher than that in a 1.0 mm follicle in the goat ovary. For reproductive hormones, the expression of *BMPR1B* was directly proportional to the increase in E${}_{2}$ content (Paradis et al., 2009). A large number of studies have shown that the *BMPR1B* gene plays an active role in mammalian embryo development and follicular development (Tang et al., 2019; Yao et al., 2019; Abdurahman et al., 2019; Zhao et al., 2016). However, further studies are required to deeply investigate the relationship between *BMPR1B* and mammalian reproduction.

### 4.2 Relationship between *BMPR1B* mutation and litter size in sheep

According to their analysis of litter size records from Booroola Merino sheep, Piper and Bindon (1982) reported that the exceptional fecundity of this breed may in part result from the action of a single major gene affecting the ovulation rate. In 1989, the Sheep and Goat Genetic Nomenclature Committee termed this gene “*FecB*” (fecundity Booroola). This point mutation A746G was in the coding region of the *BMPR1B* gene (Souza et al., 2001). To date, the *BMPR1B* mutation (*FecB*) has been found in various sheep breeds, such as Garole sheep (India; Davis et al., 2002; Polley et al., 2010), Javanese sheep (Indonesia; Davis et al., 2002), and Bayanbulak sheep (China; Zuo et al., 2013). Moreover, the polymorphism of the *BMPR1B* gene has also been extensively studied in other species. Darwish (2018) found that *BMPR1B* polymorphism was significantly associated with litter size in Egyptian goats, which could significantly increase the rate of twin births. By constructing a vector with the mutant G allele of the A746G mutation, Zhao et al. (2016) found that *BMPR1B* expression in pigs was 0.5–2 times higher in multiple tissue types of transgenic-positive F1 piglets than in their transgenic-negative siblings. Moreover, with further study of *BMPR1B*, more polymorphic sites have been found: Dutta et al. (2014) found a new mutation G773C in the Assam hill goat that may have a completely different mutation effect to A746G (*FecB*). Furthermore, mutation g.66496G>A in exon 8 of the *BMPR1B* was found in prolific Lori-Bakhtiari ewes and was reported to be significantly related to litter size (Abdoli et al., 2018).

*BMPR1B* has been identified as a major gene affecting the litter size in sheep (Juengel et al., 2013; Piper et al., 1985; Davis et al., 1982). Therefore, determining other SNPs is of great significance for improving sheep reproductive performance. In this study, we found that the allele frequencies of the g.29380950G>A, g.29380965A>G, and g.29401381C>T loci was significantly different between monotocous and polytocous breeds; this means that the three loci are significantly related to lambing traits. Additionally, most ewes contained three genotypes: two g.29401381C>T-locus-only genotypes were detected in all breeds, which may have resulted from low polymorphism, and three genotypes were detected if we expanded the number of ewes examined. In addition, the three loci were not in Hardy–Weinberg equilibrium (P<0.05) in some breeds, such as g.29380950G>A in all sheep breeds except for Tan and Prairie Tibetan sheep and g.29401381C>T in the Hu sheep breed; this may have resulted from both natural and artificial selection.

Each copy of the *FecB* mutation was found to increase ovulation by 1.65 in the highly prolific Booroola Merino breed (Piper et al., 1985). This may increase the signal intensity of the signal transduction process to downstream receptors, leading to premature follicles, increased ovulation, and a subsequent increase in the litter size (Guo et al., 2018). In this study, the g.29380965A>G locus shows similar mutations that do not cause amino acid change but have a significant negative correlation with litter size in STH sheep. The statistics regarding the litter size of the three genotypes showed that the litter size associated with the GG genotype was significantly lower than that associated with the AA and AG genotypes. Association analysis between the combined genotypes of the g.29380965A>G locus and *FecB* and litter size in STH sheep showed that the liter sizes associated with the AG–GG, AA–GG, and GG–GG genotypes were significantly higher than that associated with other genotypes; this may be due to the locus attenuating the downstream signal intensity of the BMP/SMAD signaling pathway, thereby weakening the effect of the *FecB *gene in the ovary of STH sheep, reducing ovulation, and decreasing the litter size. However, further studies are required to deeply investigate the molecular mechanism at the cellular level. Nevertheless, this study indicated that the g.29380965A>G locus may be the key locus affecting the litter size of STH sheep; thus, it could be used for selective breeding for an increased litter size in sheep.

## 5 Conclusion

In this study, we found that the *BMPR1B* gene was mainly expressed in the brain, cerebellum, hypothalamus, ovary, uterus, and oviduct of STH sheep and that the expression in the hypothalamus of the polytocous group was significantly higher than that in the monotocous group. We analyzed the population genetics of three SNPs in *BMPR1B* using association analysis and found that one key locus (g.29380965A>G) can significantly reduce the liter size in STH sheep. Further analysis indicated that this locus influenced litter size in ewes by effecting *FecB*.

## Data Availability

The data sets used in this paper are available from the corresponding author upon request.
